# Anticipating Z-generation tourists’ green hotel visit intention utilizing an extended theory of planned behavior

**DOI:** 10.3389/fpsyg.2022.1008705

**Published:** 2022-12-05

**Authors:** JiaLiang Pan, Yi-Man Teng, Kun-Shan Wu, Ting-Chung Wen

**Affiliations:** ^1^School of Business and Management, Jiaxing Nanhu University, Jiaxing, China; ^2^College of Economics and Management, Yango University, Fuzhou, China; ^3^Department of Business Administration, Tamkang University, Taipei, Taiwan

**Keywords:** personal moral norms, environmental concern, attitude, subjective norms, perceived behavior control, intention, green hotel

## Abstract

Due to the effect COVID-19 epidemic, promoting green consumption is now a key marketing strategy in the hospitality and tourism industry. As it is vital green hotels predict their customers’ visit intention, this study attempts to discover the factors affecting Taiwan’s Z-generation tourists’ green hotel visit intention using an extended theory of planned behavior [including personal moral norms (PMN) and environmental concern (EC)]. Data were gathered from 296 Z-generation tourists *via* an online survey, which was subsequently analyzed using partial least squares structural equation modeling. The results evidence that Z-generation tourists’ attitude, subjective norms, (SN) and perceived behavioral control positively and significantly influence their green hotel visit intention, with attitude being the most significant factor. Moreover, the mediation model analysis indicates Z-generation tourists’ attitude toward green hotels mediates the relationships between PMN, SN, EC, and visit intention. This study provides new insights into tourists’ green hotel visit intention and emphasizes the importance of attitude in the formation of intention.

## Introduction

**Figure 1:**
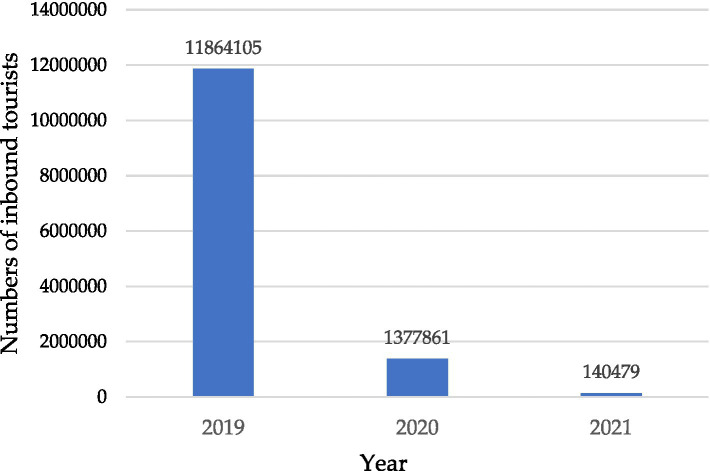
The distribution of inbound tourists from 2019 to 2021 (Taiwan Tourism Bureau, 2022).

The COVID-19 epidemic has been badly hit every field of industry globally, with the hospitality industry being one of the most severely affected. The hotel industry of Taiwan has also been severely affected. In 2020, the first year of the COVID-19 pandemic, the overall number of inbound tourists staying at travel accommodation in Taiwan fell to around 1.37 million (Taiwan Tourism Bureau, 2022). By comparison, nearly 11.8 million tourists stayed at travel accommodation in Taiwan in 2019 (Figure 1).

Furthermore, global climate change, high resource consumption, increasing pollution, and ozone depletion have reinforced the need for businesses to pay attention to global environmental issues. Today, consumers are more aware of these problems and demand hotels and hotel management establish ‘green awareness’ (Yi et al., 2018). Greater interest from consumers regarding green behaviors has led the hospitality industry to practice more environmentally friendly activities. Green hospitality offers a distinctive service-scape that inspires ecologically friendly and socially responsible behaviors (Balaji et al., 2019).

Due to the increasing public demand for environmentally friendly practices and in response to their guests’ concerns regarding the environment, many hotels are devising green strategies. Hotels worldwide have implemented various everyday green environmental practices to address both consumer pressure and the increase in environmental laws (Agag and Colmekcioglu, 2020). Hotel operators are adapting to these ‘green waves’ by incorporating environmental attributes into their services and turning their operations into “green hotels” (Verma and Chandra, 2016).

The hotel business is widely regarded as being among the most energy- and resource-intensive industries. Hotel facilities are among the top five in terms of energy usage in the commercial building sector, according to the United States Energy Information Administration (2018). This means the general business activities of the hotel industry consume vast amounts of non-renewable resources, such as water and power, and disposable products (e.g., toothbrushes, combs, razors, soap, shower caps, shampoo, and bath and shower gel; Verma and Chandra, 2018; Mak and Chang, 2019). Furthermore, Dhirasasna et al. (2020) show that the hotel sector contributes significantly to emissions of greenhouse gases. Hotel operators are now more focused on maintaining a balance between environmental problems and resource consumption (Kim et al., 2019; Wu and Cheng, 2019). Hotel operators are implementing a pro-environmental perspective into green strategies, management, and marketing activities to lessen their negative environmental effects and obtain distinct competitive benefits (Mak and Chang, 2019; Nilashi et al., 2019). Many hotels follow ecological movements, for example, the Ritz-Carlton decreased its water consumption by utilizing high-efficiency plumbing fixtures and use recycled materials including sustainable leather, wood, and building waste. Such environmentally friendly practices assist both the hotel sector and the environment (Eid et al., 2021).

With the rising awareness of ecological sustainability and environmental protection from consumers, sustainable tourism is now one of the most on-trend travel options (Merli et al., 2019; Agag and Colmekcioglu, 2020), thus focusing on environmental concerns (ECs) is imperative to satisfy eco-minded tourists. According to the 2019 annual global sustainable tourism survey report (Booking.com, 2019), roughly 80% of Taiwanese tourists support sustainable tourism, and nearly 89% of tourists expressed they would choose at least one environmentally friendly hotel for their journey. This survey shows that Taiwanese tourists have a high acceptance toward staying in environmentally friendly hotels, and their awareness of sustainable tourism continues to increase.

Consumers’ ECs and the impact these have on their green hotel selection and purchasing behavior is significant in hospitality literature. According to Arun et al. (2021) system review study, the extant literature addressing green hotel adoption outcomes classifies tourists’ behavior into five types: intention to visit (Nimri et al., 2020a,b; Yeh et al., 2021), willingness to pay premium (Yadav et al., 2019; Yarimoglu and Gunay, 2020), engaging in word-of-mouth (Wang et al., 2018; Gao, 2020), intention to take part in hotel sustainability initiatives (Teng et al., 2018), and satisfaction and loyalty (Yarimoglu and Gunay, 2020). Additionally, theoretical studies examining how guests choose green hotels utilizing various hypotheses: the theory of planned behavior (TPB; Yeh et al., 2021), the norm-activation-theory (Yan and Chai, 2021), Schwartz’s values theory (Rahman and Reynolds, 2019), social identity theory (González-Rodríguez et al., 2020), goal-framing theory (Wang et al., 2022), and the value-belief-norm theory (Choi et al., 2015).

Although multiple theoretical perspectives are utilized to understand an individual’s green hotel visit intention, TPB is the most commonly used theoretical model to predict consumers’ green hotel visit behavior (Teng et al., 2015, 2018; Ting et al., 2019; Arun et al., 2021). The TPB (Ajzen, 1991) has been used in over 2000 empirical investigations of green behavioral science (Ajzen, 2019), and Ulker-Demirel and Ciftci (2020) believe that the TPB is appropriate for studying customers’ green behavior and behavioral intention (BI). The TPB theory is also regarded as a leading framework as it has great applicability when explaining consumers’ green hotel visiting behavior and in environmental psychology research (Yadav et al., 2019).

Due to the scarcity of environmental resources, one person’s consumption will be at the expense of another’s interests. Thus, some researchers believe that, to a certain extent, green purchasing behavior has moral commitment (Botetzagias et al., 2015). Furthermore, several studies argue that personal morals should be applied to the TPB for investigating green purchasing behavior (Botetzagias et al., 2015; Chen, 2016; Liu et al., 2020), and state personal moral norms (PMN) should be applied to the TPB as they are generally perceived to improve the predictability of original TPB structures (Chan and Bishop, 2013).

PMN are prompted by situational cues when an individual’s value system “*generates feelings of moral obligation to perform or refrain from specific actions*” (Biel and Thøgersen, 2007). Some research findings provide extended support for applying PMN to the TPB model when predicting the intention of pro-environmental behavior (Shi et al., 2017; Ru et al., 2019). Additionally, there are literature that evidence the incorporation of perceived moral obligation and moral reflectiveness to the TPB increases the predictive ability of green hotel visit intention within the proposed conceptual TPB model (Verma and Chandra, 2018). Therefore, this study predicts that PMN is a crucial factor affecting consumers’ willingness to choose green hotels.

In addition, findings from prior studies suggest that ECs have a significant impact on consumers’ ecological intents and behavior (Chan et al., 2017; Balaji et al., 2019). EC is often expounded as “*the person holds values, attitudes, emotions, perceptions, knowledge, and behaviors regarding environmentally related activities or issues*” (Ogle et al., 2004; Schultz et al., 2004). According to recent empirical literature, incorporating EC into the TPB model elicits a greater understanding of consumers’ green tourism behavior, including green peer-2-peer (P2P) accommodation (Agag, 2019), hotel guests’ water conservation behaviors (Han and Hyun, 2018), and tourists’ slow decision-making processes (Meng and Choi, 2016). These findings provide sufficient evidence to verify customers with strong environment concerns are more supportive attitude of green tourism practices, which increases customers’ intent to stay in green hotels (Chen and Tung, 2014). EC has always been regarded as an important predictor of ecological behavior (pro-environmental behavior) and decision-making; thus, this article argues EC is an essential factor affecting consumers’ tendency to select green hotels.

Even though the notion of green hotels has been widely researched from a Western perspective, it remains short of a coherent basis. In addition, the current research on individual’s preference for green hotels is mainly in developed countries and does not take into account the contribution of consumers in developing countries. Therefore, the objective of this study is twofold. First, by combining PMN and EC into the TPB framework, this research aims to close the gap by predicting Generation Z visitors’ intention for green hotels. Generation Z individuals are born after 1995, with the majority of them yet to enter the workforce (Cilliers, 2017). Many businesses have identified Generation Z as their new short-term target market because of their higher propensity to engage in social and economic activity (Song et al., 2020). Therefore, understanding the contributing factors in the decision-making process for Generation Z to visit green hotels is an important insight for future green hospitality development. The second aim is to explain how the PMN, EC, and TPB models forecast the guest visiting intention for green hotel.

Our study makes some contributions to literature, theory, and managerial implications. The fundamental contribution of this study, which is based on the TPB model, PMN, and EC, aims to increase our understanding of the field of green hotel visits. Explicitly, this study empirically tests and supports the association among the concept of PMN and EC and key factors of TPB in the development of visit intention toward green hotels. This work presents particular practical implications for green hotel practitioners in addition to theoretical contributions, as the hotel requires eco-friendly management to attain a competitive edge. Furthermore, this study adds to the body of knowledge regarding green hotel visiting intentions in the context of hotel operating environments to come and its potential advantages for achieving the sustainable development goals (SDGs), especially those linked to SDGs 6 (clean water and sanitation), SDG 7 (affordable and clean energy), and SDG 12 (responsible consumption and production).

## Literature review

### Role of the TPB in green hotels

Ajzen (1991) TPB, as a theoretical framework, states that individual behavior is motivated by BI, which are a function of three individual factors: attitude (AT), subjective norms (SN), and perceived behavioral control (PBC), that would contribute significantly to both theory and practice in hospitality management research (Ulker-Demirel and Ciftci, 2020). Han et al. (2010) pioneered the use of the TPB to anticipate consumers’ green hotel visit intention. In hospitality studies, the TPB (Fishbein and Ajzen, 1975; Ajzen and Fishbein, 1980) is the most applied theory to understand consumer adoption behavior toward green hotels, with 24 of the 76 studies utilizing the theory in green hotel literature (Arun et al., 2021).

Lately, many articles have tried to incorporate the TPB model into the study of hotel pro-environment behavior. Numerous papers studied the role of three TPB constructs in intention to stay in green hotel (Verma and Chandra, 2018; Wang et al., 2019; Nimri et al., 2020a,b; Bouarar et al., 2021; Yeh et al., 2021; Fauzi et al., 2022; Kim and Ha, 2022). Prior TPB literature toward green hotel visit intention declare that consumers’ attitude toward green hotels and the degree to which a person appraises green hotels primarily guide their BI to visit them (Jiang and Gao, 2019; Eid et al., 2021). Evidence of SN significantly affects an individual’s BI, which indicates individuals value how important it is to their relevant reference group to visit a green hotel (Teng et al., 2015; Yarimoglu and Gunay, 2020). Previous studies also prove that consumers’ PBC directly affects intention to stay in a green hotel (Verma and Chandra, 2018; Yadav et al., 2019). These three original TPB variables significantly support consumers’ adoption of green hotel products and services.

In extant literature, several internal mental variables are studied as antecedents to TPB green hotel behavior. These are classified as cognitive psychology, environmental psychology, and value beliefs. In cognitive psychology, environmental knowledge, green hotel knowledge, and low-carbon knowledge have a positive influence on the consumer’s decision-making processes regarding their green hotel visit intention (Nimri et al., 2017; Teng et al., 2018; Wang et al., 2022). In environmental psychology, sacrifice for the environment, EC, environmental attitude, environmental consciousness, and environmental value determine consumers’ green hotel decision-making processes (Huang et al., 2014; Rahman and Reynolds, 2016; Teng et al., 2018; Bashir et al., 2019; Patwary et al., 2021). In value beliefs, beliefs, moral effectiveness, religiosity, altruism, biospheric values, hedonism, collectivistic values, moral obligations, personal norms, and green self-identity exert a positive influence on an individual’s green hotel visit intention (Yadav et al., 2019; Agag and Colmekcioglu, 2020; Fatoki, 2020; Yeh et al., 2021). These results evidence that both BI and attitude can be influenced by external factors, which contributes an alternative perspective to the extant green hospitality literature.

Recently, Olya et al. (2019) conducted a study on green hotels, verifying that the younger generation may choose such hotels in order to protect the environment as much as possible. As a result, Mugiarti et al. (2020) used the TPB framework to predict young customers’ intention toward green hotels in Indonesia. Surprisingly, given the unprecedented potential of the green hotel market in Taiwan, there has been little research into the green hotel visiting behavior of young people (Z-generation) in the region.

### Relationship among TPB variables

Wang et al. (2022) reveal that the TPB constructs of consumers’ attitude, SN, and PBC positively influence consumers’ green hotel visit intention. Previous study has also found a substantial association between attitude, intention, and behavior, confirming the theoretical nexus between the two components in the context of green hotel consumers (Agag and Colmekcioglu, 2020). Attitude is the assessment of a given behavior that is depending on the perceived advantages and disadvantages of the behavior of the behavior (Lindenberg and Steg, 2007; Wang et al., 2020). SN are influenced by social pressure from friends, relatives, and co-workers that cause an individual to perform certain or expected behaviors (Ajzen, 1991). Ulker-Demirel and Ciftci (2020) state SN inspire consumers to transform their green products’ behavior at a diverse macro-level setting. As SN significantly influence an individual’s sustainable behavior, consumers’ friends or relatives possess the ability to encourage or discourage an individual’s green hotel visit intention (Ting et al., 2019).

PBC indicates whether a particular behavior is perceived as difficult or easy by the consumer (Ajzen, 1991). In light of several studies, PBC positively influences intention to visit green hotels and as a result, also has a significant effect on individuals’ green hotel visit intention (Verma and Chandra, 2018; Yadav et al., 2019). The article critically examines the application of the TPB to the research objective, which is to analyze intentions and attitudes of Generation Z regarding green hotels. Thus, the following hypotheses are proposed:

*H1*: Attitude toward green hotels has a significant effect on green hotel visit intention.

*H2*: Green hotel visit intention is significantly affected by SN associated with green hotels.

*H3*: Green hotel visit intention is significantly affected by PBC associated with green hotels.

*H4*: Attitude toward green hotels is significantly affected by SN.

### PMN

PMN, as defined by Schwartz (1977), are “*an individual’s belief that acting in a certain manner is right or wrong because he feels a moral norm to do so*.” Conner and Armitage (1998) propose PMN as “*one’s own socially determined and socially validated values attached to a particular behavior*.” Ajzen (1991) also proposes personal feelings of individual’s moral obligations toward BI need to be examined in some circumstances, while several other studies evidence PMN can be a useful predictor of BI (Heath and Gifford, 2002; Chan and Bishop, 2013; Botetzagias et al., 2015). Researchers also suggest that in the context of consumer green behavior, PMN are vital predictors of consumers’ purchase attitude and BI (Thøgersen and Ölander, 2006; Si et al., 2020). Contrastingly, there are studies that demonstrate engaging PMN significantly influences individuals’ eco-friendly intentions or behavior, such as an individual’s energy-saving behavior (Gao et al., 2017), recycling intention (Wan et al., 2014), public transport use (Shi et al., 2017), and BI toward reducing PM2.5 (particulate matter 2.5; also classed as moderate air pollution; Ru et al., 2019). Previous literature evidence PMN has a more effective interpretation ability in predicting individuals’ green behavior than the original TPB (Gao et al., 2017; Liu et al., 2020). Additionally, some literature empirically prove PMN represent the dominant cognitive constructs in the TPB and explain consumers’ green hotel visit intention (Verma and Chandra, 2018; Liu et al., 2020).

In summary, the aforementioned articles propose that heightened PMN predisposes consumers green behavior, as this provides individuals with the opportunity to accomplish the required PMN motives desired by society. Hence, the following hypotheses are proposed:

*H5*: PMN positively affect intention toward green hotels.

*H6*: PMN positively affect attitude toward green hotels.

*H7*: The relationship between PMN and green hotel visit intention is mediated by AT.

### EC

EC is defined as “*the awareness about the environmental problems and the willingness of a person to be a part of the solution*” (Dunlap and Jones, 2002), and “*a form of personal awareness of environmental issues and the desire to take concrete ecological sustainability actions*” (Shin et al., 2017). Previous research evidence EC as a vital predictor of environmentally friendly green products purchase behavior (Zhang et al., 2019). Additionally, EC is recognized as a dominant cognitive construct to predict green restaurant visit intention (Hu et al., 2010; Teng et al., 2014). Similar results indicate that increased EC also leads to increased green hotel visit intention (Agag, 2019; Jiang and Gao, 2019). Wang (2022) also found that higher EC always leads to an increase in consumers’ purchasing attitudes and intentions regarding staying at green hotels. That is, the level of consumers’ consciousness toward environmental issues influences their attitude toward green hotels and consequently, the emergent related behavior.

Paul et al. (2016) propose that EC, as a construct, must be included in the TPB model to obtain a deeper understanding of customers’ intentions to make green purchases. Other studies combine the EC variable into the TPB framework and have found EC has a significant influence on consumers’ green product attitudes, SN, PBC, and buy intentions for a variety of green items (Zhang et al., 2019). In the hospitality context, several scholars also combine EC as an antecedent to the TPB framework and evidence that the greater the EC, the more positive the effect on staying green hotel intention (Chen and Tung, 2014; Agag, 2019). Based on the literature review, this research discusses the direct effect EC has on the TPB constructs, as well as its mediating effect. Thus, the final three hypotheses are:

*H8*: EC is positively correlated with the intention toward staying in a green hotel.

*H9*: EC is positively correlated with the attitude toward staying in a green hotel.

*H10*: The association between EC and green hotel visit intention is mediated by AT.

## Research methodology

### Questionnaire development

**Figure 2:**
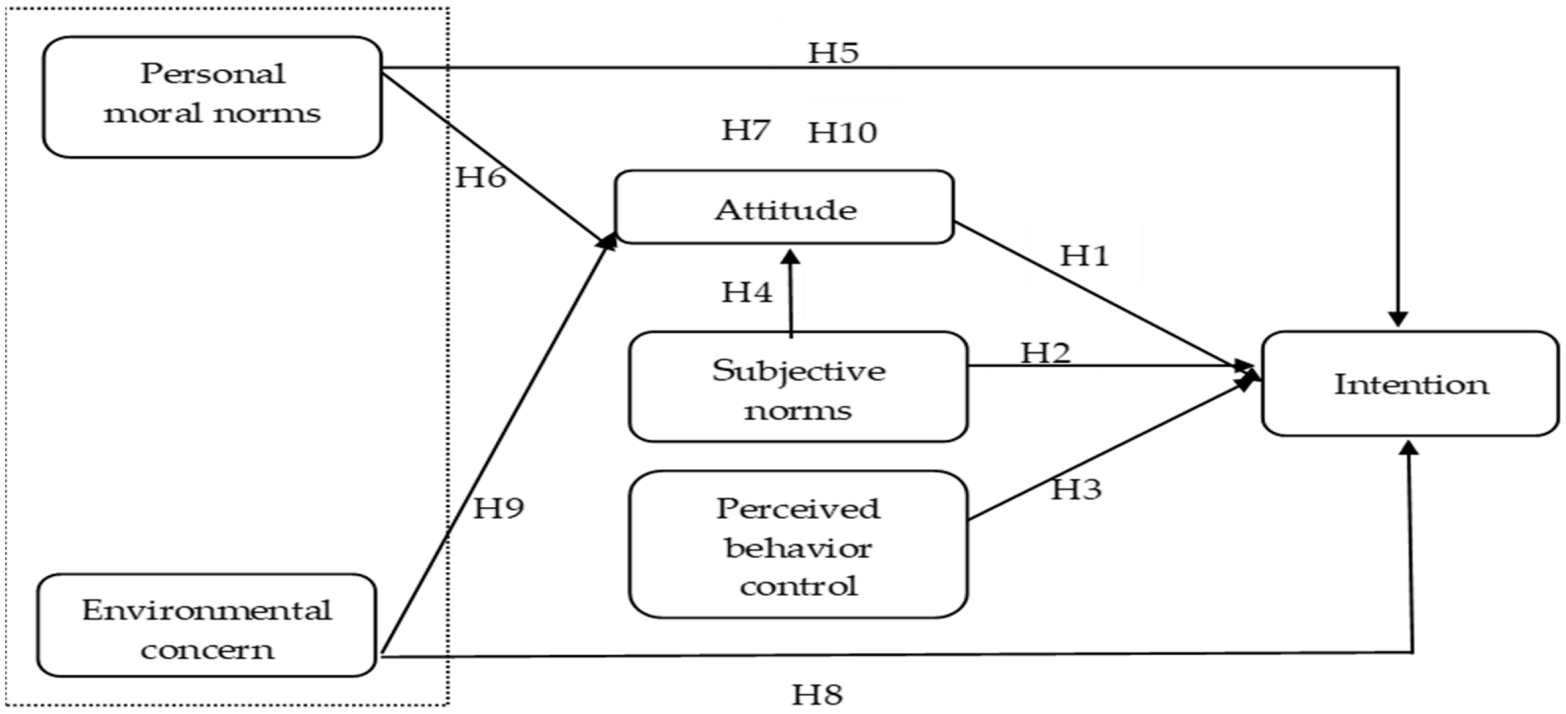
Theoretical framework.

The purpose of this paper is to explore Generation Z’s BI toward visiting green hotels. The conceptual framework is empirically tested *via* integrating PMN and EC into the TPB using a consumer survey completed by Generation Z (Figure 2). The survey design is predominantly based on the research framework shown in Figure 2. All measures are derived from relevant studies where they have been previously validated. There are, however, minor changes to the wording to ensure the questionnaire is understandable with regard to visiting green hotels.

The measurement items contain questions relating to the combination model and include six constructs: PMN, EC, attitude (AT), SN, perceived behavior control (PBC), and green hotel visit intention (INT). The construct questions are measured using a 7-point Likert-type scale. The higher the score, the higher the level of agreement or conformity. Appendix 1 shows the item details that measure sustainable use behavior relating to green hotels and the reference sources.

### Data collection and sample description

This study’s sample comprised Taiwanese tourists who were 18 years of age or older and were inclined to travel and stay in green hotels in the future. A pilot study was done in December 2019 prior to the main study. The pilot test was validated by interviewing two experts and the questionnaire was shared with 36 undergraduate and graduate students from the author’s institution to assess its reliability. It was determined from the pilot tests that each item had adequate reliability because they were all above the crucial value of 0.7 (Nunnally and Bernstein, 1994). As a result, we continued with the questionnaire to gather data and did not eliminate any questions from the constructions.

The complete survey was distributed online from January to March 2020 after the pilot study’s improvements. The questionnaire (see Appendix 1) was conducted using an online survey *via* Line using a Google Forms link. The survey was completely anonymous and informed consent was obtained from the participants.

A total of 296 valid responses were obtained. As it was impractical to estimate the size of the parent body, employing the calculation formula suggested by Bateman et al. (2002) with a 95%confidence interval gave an effective sample size of 249. As 296 valid responses were recovered this should be representative of the parent body (Lin et al., 2022).

### Data analysis

According to Hair et al. (2012a,b) whether a research constructed theoretical model is exploratory or expansible, it is more suited to partial least squares structural equation modeling (PLS-SEM) as it has stronger predictability compared to covariance SEM. Therefore, PLS-SEM is used for the empirical analysis in this study, which is carried out in two stages. First, the measurement model is assessed to ensure the effectiveness and reliability of the construct. Second, the structural model is checked to validate the research hypothesis.

## Results

### Demographic analysis

The demographic variables of the respondents collected in this study is in line with the previous articles regarding the green hotel visiting intention (Han et al., 2011; Nimri et al., 2020a; Dwivedi et al., 2022). The 296 respondents’ specific demographic distribution is listed in Table 1. Of the total respondents, 66.9% of participants are male and 33.1% female. The percentage of participants born 1995–1999 is 51.4%, and 2000–2002 is 48.6%. Most participants have a bachelor’s degree (81.4%).

**Table 1:** Summary of descriptive statistics.

Characteristic	Demographic	Frequency	Percentage (%)
Gender	Male	198	66.9
	Female	98	33.1
Year born	1995–1999	152	51.4
	2000–2002	144	48.6
Education	Undergraduate	241	81.4
	Master student	19	6.4
	Bachelor’s degree	22	7.4
	Master’s degree	14	4.8

### Measurement model assessment

The measurement model was estimated using confirmatory factor analysis. Factor loading, Cronbach’s alpha, composite reliability (CR), and average variance extracted (AVE) were used to evaluate convergent validity (Table 2).

**Table 2:** Confirmatory factor analysis.

Variable	Item	Mean	SD	FL	CR	AVE	CA
Personal moral norms	PMN1	5.118	1.142	0.841	0.896	0.682	0.843
	PMN2	4.865	1.154	0.863			
	PMN3	5.041	1.207	0.777			
	PMN4	4.328	1.209	0.821			
Environmental concern	EC1	5.797	1.158	0.788	0.899	0.689	0.845
	EC2	6.024	1.013	0.869			
	EC3	5.902	1.064	0.799			
	EC4	5.959	0.994	0.862			
Attitude	AT1	5.233	1.192	0.877	0.957	0.815	0.943
	AT2	5.071	1.172	0.921			
	AT3	4.838	1.176	0.887			
	AT4	4.628	1.212	0.904			
	AT5	4.784	1.179	0.924			
Subjective norms	SN1	4.378	1.340	0.951	0.952	0.868	0.924
	SN2	4.328	1.339	0.954			
	SN3	4.679	1.255	0.888			
Perceived behavioral control	PBC1	5.034	1.170	0.879	0.880	0.712	0.803
	PBC2	5.385	1.230	0.756			
	PBC3	4.885	1.262	0.890			
Intention	INT1	5.142	1.165	0.893	0.921	0.796	0.871
	INT2	4.770	1.191	0.910			
	INT3	4.618	1.281	0.873			

After PMN5 was deleted, all factor loadings exceeded 0.50, the values of Cronbach’s α and CR exceeded the minimum requirements of 0.70, and AVE values exceeded the threshold of 0.50 (Yusof et al., 2012; Hair et al., 2017), representing the convergent validity is satisfactory.

By comparing the square root of AVE values with the correlations between constructs, discriminant validity was evaluated for the correlation between variables and constructs (Fornell and Larcker, 1981). The results of examining those constructs indicate the discriminant validity is satisfactory (Table 3).

**Table 3:** Fornell-Larcker discriminant validity assessment.

Construct	PMN	EC	AT	SN	PBC	INT
PMN	0.682					
EC	0.251**	0.689				
AT	0.566**	0.218**	0.815			
SN	0.352**	0.116**	0.590**	0.868		
PBC	0.232**	0.135**	0.277**	0.252**	0.712	
INT	0.464**	0.210**	0.676**	0.497**	0.342**	0.796
Mean	4.838	5.921	4.911	4.462	5.101	4.844
SD	0.972	0.875	1.071	1.222	1.034	1.081

PMN, personal moral norms; EC, environmental concern; AT, attitude; SN, subjective norms; PBC, perceived behavior control; INT, intention; **denotes *p* < 0.01.

The collinearity must be checked prior to evaluating the structural model, as path coefficients may be biased if a significant level of collinearity exists between the different latent variables (Hair et al., 2012a,b). In the model, the full variance inflation factors of all structures are smaller than 3, showing that do not exist multi-collinearity concerns (Hair et al., 2011). Additionally, the common method bias is not regarded as an issue (Kock and Lynn, 2012).

### Hypothesis tests

**Figure 3:**
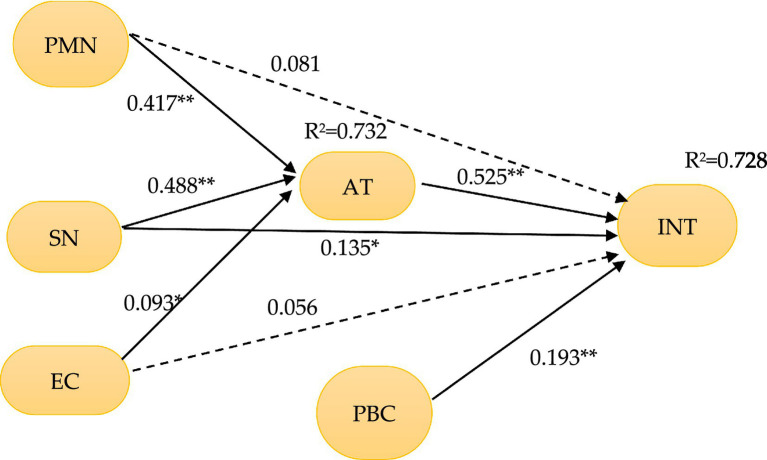
Measurement and structural model. (1) PMN, personal moral norms; EC, environmental concern; AT, attitude; SN, subjective norms; PBC, perceived behavior control; INT, intention; (2) a solid line represents a significant path, and a dashed line represents an insignificant path; (3) *p < 0.05, **p < 0.01.

The suggested model was tested using 5,000 bootstrap resamplings to determine the significant path coefficients (Li et al., 2022), and to validate all hypotheses. Table 4 contains the relevant path coefficient, t-value, and value of p, and the outcomes are given in Figure 3.

**Table 4:** Results of measurement model testing.

Hypothesis	Path	Path coefficient	Standard error	*t*-value	Hypothesis support
H1	AT➔INT	0.525**	0.527	7.210	Yes
H2	SN➔INT	0.135*	0.136	2.406	Yes
H3	PBC➔INT	0.193**	0.195	3.975	Yes
H4	SN➔AT	0.488**	0.487	10.225	Yes
H5	PMN➔INT	0.081	0.085	1.805	No
H6	PMN➔AT	0.417**	0.419	8.672	Yes
H7	EC➔INT	0.056	0.052	1.667	No
H8	EC➔AT	0.093*	0.093	2.233	Yes

*p < 0.05, **p < 0.01.

PLS-SEM can be used to obtain the determinant coefficient R^2^, that is, the amount of variance explained by the independent variable, indicating the explanatory ability (Fernando and Hor, 2017). On the basis of Hair et al. (2012a,b) studies, *R*^2^ value of 0.2 or above has a higher predictive and explanatory power. In this study’s model, the *R*^2^ value of green hotel visit intention is 0.728, which indicates the proposed model has good predictive accuracy.

The findings of all hypothesis verification are shown in Table 4. AT significant positive impact INT (*β* = 0.525, *T* = 7.210, *p* < 0.01), meaning H1 is supported. SN significant positive impact INT (*β* = 0.135, *T* = 2.406, *p* < 0.05), which means H2 is supported. PBC significantly positively effect on INT (*β* = 0.193, *T* = 3.975, *p* < 0.01), meaning H3 is approved. AT is positively impacted by SN which means H4 is supported (*β* = 0.488, *T* = 10.225, *p* < 0.01). H5 is not confirmed because the impact of PMN on INT is insignificant (*β* = 0.081, *T* = 1.805, *p* > 0.05). H6 is confirmed since the impact of PMN on AT is significant (*β* = 0.081, *T* = 1.805, *p* > 0.05). H8 is not confirmed since the impact of EC on INT does not have a significant influence (*β* = 0.056, *T* = 1.667, *p* > 0.05). Lastly, EC significantly positively effect on AT (*β* = 0.093, *T* = 2.233, *p* < 0.05), meaning H8 is supported. Overall, six of the eight hypotheses are supported, with the effects of PMN and EC on INT being the only ones that are not statistically significant.

### Mediating effects

To test the mediating effect of AT, the bootstrapping analysis (5,000 bootstrap samples with 95% confidence intervals) was conducted. Table 5 shows that PMN (*β* = 0.2189, *p* < 0.05; 95% CI = [0.5323, 0.7393]), SN (*β* = 0.2562, *p* < 0.05; 95%CI = [0.3759, 0.5650]), and EC (*β* = 0.0488, *p* < 0.05; 95%CI = [0.3440, 0.5717]) have significant positive indirect influences on INT through AT. Such effects are confirmed by the corresponding value of *p*s (*p* < 0.05), with 95% confidence intervals where the upper and lower limits do not include zero.

**Table 5:** The bootstrap results of the mediation.

Indirect effect	Estimate	Standard error	95% bias corrected CI	
			Lower	Upper
PMN → AT→INT	0.2189	0.0525	0.5323	0.7393*
SN → AT→INT	0.2562	0.0480	0.3759	0.5650*
EC → AT→INT	0.0488	0.0576	0.3440	0.5717*

PMN, personal moral norms; EC, environmental concern; AT, attitude; SN, subjective norms; INT, intention; *denotes *p* < 0.05.

### Further analysis

Despite being gathered in practically all quantitative studies, gender, age, and education have only been considered as the control variables in a few studies (Arun et al., 2021). Therefore, this study included demographic variables as control variables to explore whether customers’ intention toward a green hotel differ across gender, age, education, and household income.

**Figure 4:**
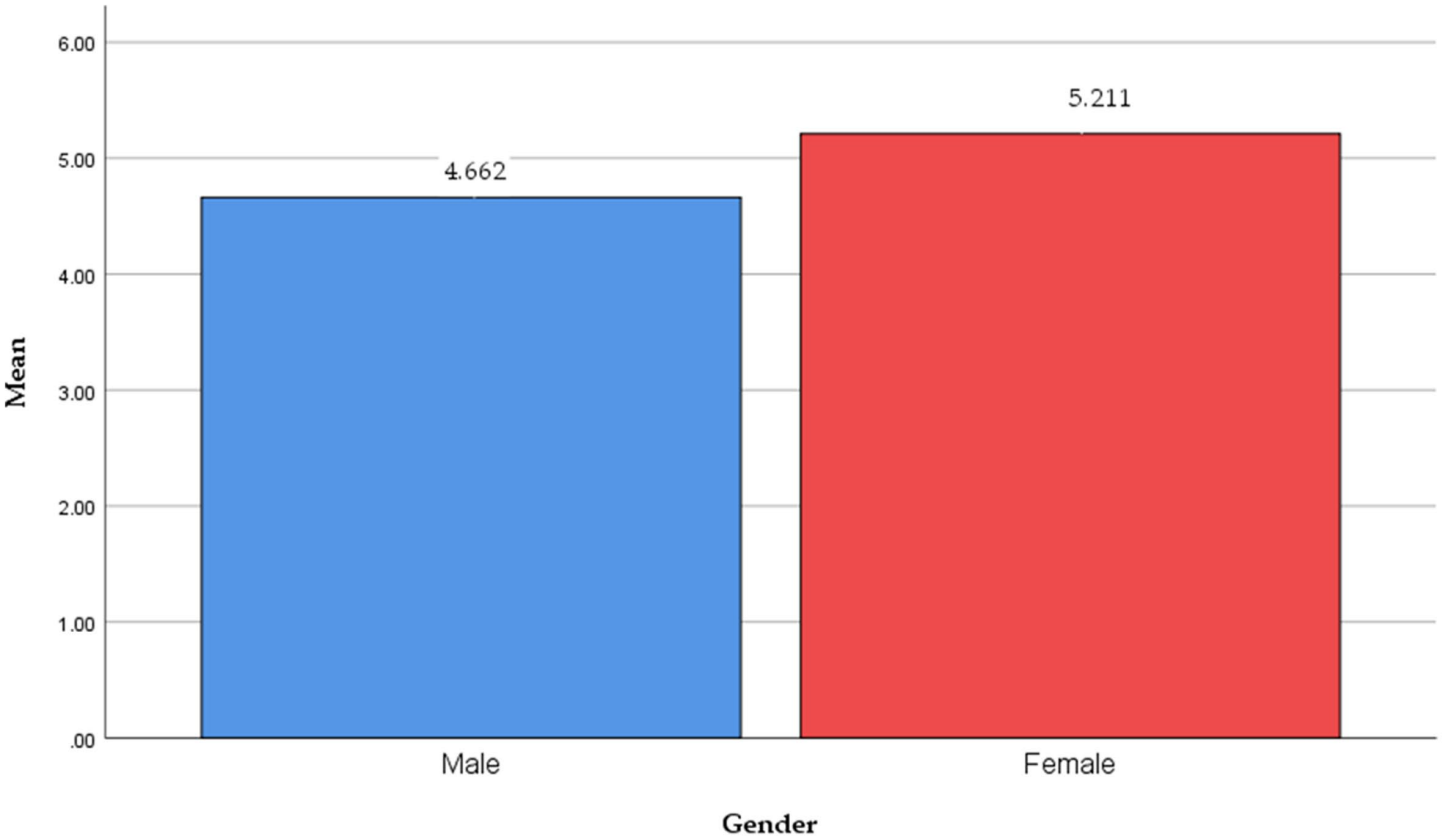
Gender difference in intention.

First, the independent T-test exposed significant differences in intention across gender groups (*t*-value = −4.229, *p* = 0.000). Table 6 and Figure 4 present the results of the independent T-test. The female group had greater mean scores for green hotels visiting intentions than the male group (*M*_female_ = 5.211 vs. *M*_male_ = 4.662). These findings implied that female tourists showed greater willingness to visit a green hotel. This finding agrees with the propositions of Han et al. (2011) that gender differences in travelers’ intentions to stay at green hotels.

**Table 6:** Results of demographic differences in intention.

Characteristic		Mean (SD)	*t*/*F* value	Value of *p*	Comparisons
Gender	Male	4.662 (1.129)	−4.229**	0.000	(a) < (b)
	Female	5.211 (0.873)			
Age	Born 1995–1999	4.910 (1.043)	1.089	0.277	
	Born 2000–2002	4.774 (1.120)			
Education	Undergraduate^(a)^	4.813 (1.064)	3.139*	0.026	(b) < (c)
	Master student^(b)^	4.526 (1.172)			
	Bachelor’s degree^(c)^	5.470 (0.852)			
	Master’s degree^(d)^	4.810 (1.319)			

*t*, Student’s *t*-test; *F*, analysis of variance (ANOVA) test. *SD*, standard deviation; *p < 0.05; **p < 0.01; multiple comparisons between each two categories are done by *post-hoc* analysis (Fisher’s least significant difference). ^a^ represents the “Undergraduate” group; ^b^ represents the “Master student” group; ^c^ represents the “Bachelor’s degree” group; ^d^ represents the “Master’s degree” group. “(a)<(b)” should be “Male < Female”.

**Figure 5:**
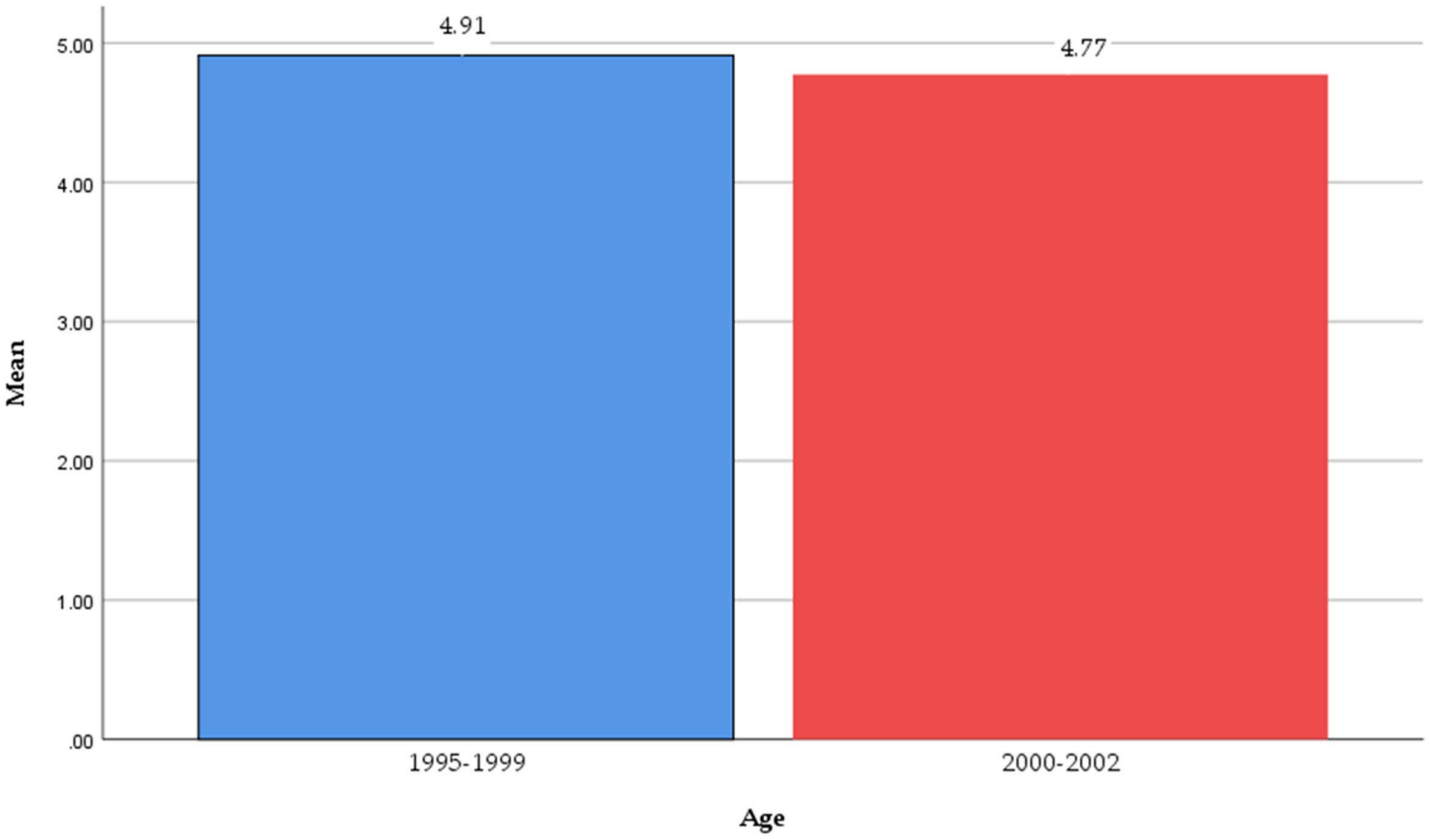
Age difference in intention.

Second, the results evidenced that mean scores in intention for the post-2000s generation group were slightly lower than other age groups (*M*_2000–2002_ = 4.774 vs. *M*_1995–1999_ = 4.910). Nevertheless, as presented in Table 6 and Figure 5, the results of the independent T-test revealed that intention was not statistically significantly different among age groups (*t*-value = 1.089, *p* = 0.277). The results showed that travelers’ intentions to stay in green hotels were not significantly influenced by age. This finding agrees with the propositions of Han et al. (2011).

**Figure 6:**
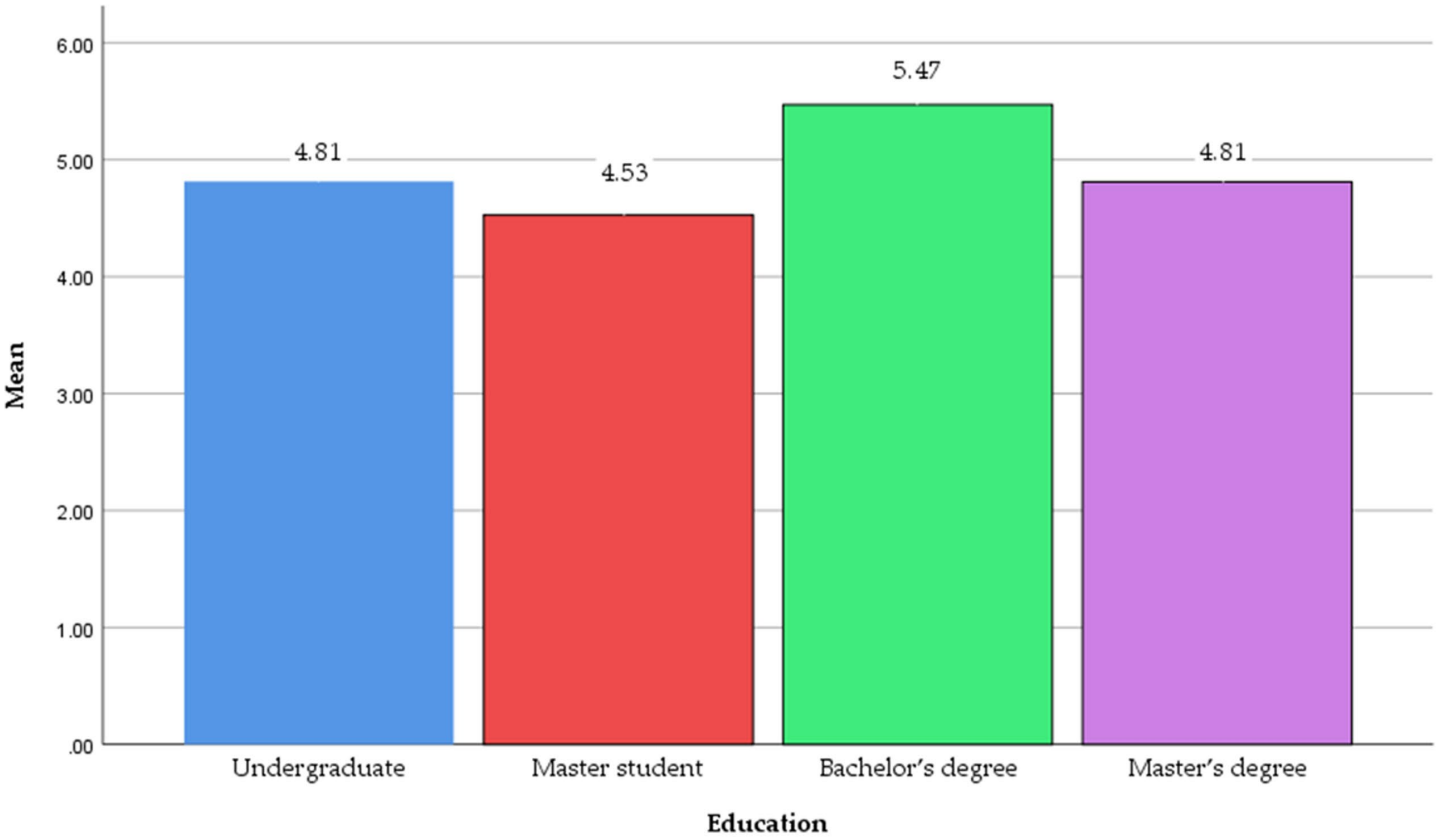
Education difference in intention.

Third, education differences in customers’ visiting intention toward a green hotel were analyzed. Table 6 exhibits the mean scores for educational groupings and Figure 6 (Ranked high to low; *M*_Bachelor’s degree_ = 5.470 vs. *M*_Undergraduate_ = 4.813, *M*_Master’s degree_ = 4.810, and *M*_Master student_ = 4.526). The “Bachelor’s degree” group indicated the highest mean values among the four education groups. ANOVA test results revealed statistically significant differences in green hotel patron intention among education levels (*F*(3, 292) = 3.139, *p* = 0.026) (see Table 6). Additionally, a *post hoc* test (Fisher’s LSD) for education groups revealed a significant difference between “Master student” and “Bachelor’s degree” groups. The results of the current study are consistent with previous research on educational differences in willingness to visit green hotels (Han et al., 2011).

## Discussion

### Findings

This study’s focal point was to integrate the TPB model with person moral norms (PMN) and EC to explore three TPB constructs (AT, SN, and PBC), PMN, and EC in understanding Taiwan Z-generation tourists’ intentions toward green hotels. In addition, the study examines the mediating role of AT in the relations between antecedent variables (PMN, SN, and EC) and the Z-generation tourists’ green hotel visiting intention.

TPB has been shown to explain individuals’ willingness to choose a green hotel (Yarimoglu and Gunay, 2020; Yeh et al., 2021). The reason why this study is based on TPB is that this theory contributes to the process of behavior, BI, and information, providing researchers with a systematic approach to identify, measure and conceptualize the determinants of behavior. Furthermore, in this study, the model was enlarged by incorporating PMN and EC, which had not previously been available or researched.

To a certain extent, the originality of this article can be observed by extending the TPB by combining PMN and EC as antecedents that would explain Taiwan Z-generation tourists’ green hotel visiting intention. Adding new variables tested in this research would help enhance TPB’s predicting power in understanding the intention toward green hotels (Yarimoglu and Gunay, 2020). As a result, the empirical results reveal multiple findings.

First, all three TPB constructs (AT, SN and PBC) strongly positive intention toward green hotels. This finding concurs with the propositions of several studies (Yadav et al., 2019; Yarimoglu and Gunay, 2020; Yeh et al., 2021). AT is the main TPB construct impacting tourist’ intention to stay in green hotels, then PBC and SN. Moreover, this research also scrutinizes the mediating influence of AT in the relations between SN and the Z-generation tourists’ green hotel visiting intention.

Second, this study confirms PMN significantly influence attitude toward green hotels. This finding concurs with Klöckner (2013), Fornara et al. (2016), and Ru et al. (2019), who found that PMN positively affect consumers’ attitude. Thus, consumers with increased PMN will likely have a significantly greater attitude toward green hotel selection. Conversely, the research results suggest there is no significant relationship between PMN and green hotel visit intention, which is not consistent with the findings of Han (2015), Rahman and Reynolds (2016), Verma and Chandra (2018), and Liu et al. (2020). The potential reason for this is the research participants are Generation Z travelers who are more individualistic and less susceptible to external secular moral values.

Third, the research outcomes reveal EC is a critical indicator of consumers’ attitude toward green hotels. This outcome is in line with prior research of Chen and Tung (2014) and Rahman and Reynolds (2016). In contrast, EC was not significant on green hotel patronage intention. Despite the literature (Agag, 2019; Wang, 2022) demonstrating the significance of EC, this study found the opposite, probably because it was conducted by members of the Z-generation.

Lastly, the findings also indicate that Generation Z consumers’ attitude toward green hotels partially mediates the influence of PMN and EC on BI. That is to say, Generation Z consumers’ PMN and EC will partially influence green hotel visit intention through their positive attitude. This finding is consistent with many prior studies’ that confirm attitude has a mediating effect on PMN, EC, and BI (Verma and Chandra, 2018; Verma et al., 2019; Liu et al., 2020).

Although PMN and EC did not have a significant direct effect on green hotel visit intention in this study, attitude is regarded as a teleological evaluation effected by perceived consequences of BI (Agag and Colmekcioglu, 2020). A favor or unfavorable attitude toward a certain behavior determines the intention to display or avoid that behavior.

### Managerial implications

#### Theoretical implications

This study provides numerous theoretical contributions to academic hospitality literature. The first part is an analysis of PMN and EC in the extended TPB model to assess Taiwanese consumers’ intention toward green hotels. This study’s findings, in line with previous research, affirm the requirement to develop and implement contemporary models to raise understanding of consumers’ green behavior within the hospitality industry (Agag and Colmekcioglu, 2020). Recently, how PMN influences consumers’ intention toward green hotels is of growing interest to researchers (Verma and Chandra, 2018; Liu et al., 2020). However, this study only demonstrates how consumers’ attitude toward green hotels has a partially mediating effect on PMN and green hotel visit intention. The results also confirm that younger consumers’ attitudes toward green hotels have a partially mediating effect on EC and green hotel visit intention. The research findings indicate that inclusion of consumers’ attitude toward green hotels as a partial mediator increases the explanatory power of younger consumer’s green hotel visit intention.

This framework provides an additional TPB model to raise understanding of consumers’ intention toward green hotels and addresses the lack of literature on the underlying mechanism. The new TPB model also provides researchers with new directions to explore other important mediators that explain how attitude toward green hotels influence consumers’ intention to visit green hotels.

Second, this research is the first to integrate these factors into the TPB for the Generation Z focus group, thus proving that individuals’ beliefs and moral norms are a contributing factor. Looking to the future, Generation Z is the worlds’ primary consumer demographic; however, currently, studies on Generation Z in the hospitality industry are sparse. This study provides evidence of an extended TPB theoretical framework to measure Generation Z’s green hotel visit intention.

#### Practical implications

From a practical standpoint, the findings provide important information for green hotel practitioners. To begin, measuring and observing tourists’ intentions to stay in green hotels are the most crucial task in the hospitality sector. To motivate tourists’ intention to visit green hotels, practitioners first need to focus on the three determinants of TPB and understand the specific beliefs behind them. Among the three determinants, attitude toward green hotels is the primary factor contributing to green hotel visit intention. Hotel practitioners and managers could enhance Generation Z’s attitude toward green hotels *via* social media communications (e.g., WeChat, TikTok, Twitter, Instagram), to increase their belief in green hotels’ environmentally friendly practices. Additionally, they could establish education programs that provide pro-environmental materials and curriculum to enhance Generation Z tourists’ knowledge of green hotel practices, thus also enhancing their attitude and belief toward green hotels. Furthermore, green hotel managers could emphasize the positive emotional experiences green hotels provide for guests during their stay, and encourage guests to share pictures, videos, and experiences *via* social media. Utilizing social media and electronic word of mouth (e-WOM) marketing strategies ensures rapid circulation of information to promote favorable and positive attitudes toward green hotels, which is crucial to increasing consumers’ intention to green hotel.

Second, the results indicate social norms have the greatest impact on attitude toward visiting green hotels. Generation Z tourists’ behavior is heavily influenced by their collectivist society, meaning friends, relatives, and colleagues help shape attitudes toward green hotels. As increased social norms determine more favorable attitudes, green hotel managers should consider e-WOM and hotel guest generated forums as vital communication channels. Development of multiple social media platforms and creating positive green hotel images promotes an impression of environmental leadership. If consumers believe in what they see, they will encourage their friends to stay in the green hotel also.

Third, PMN is a vital in affecting Generation Z tourists’ attitude toward green hotels. Reaching, engaging, and influencing tourists with PMN is a challenge for green hotels. The hoteliers should therefore invest in effective marketing to include elements that raise customer awareness of and interest in environmental issues and can draw tourists. Hotel marketers could implement promotional campaigns focusing on the importance of the eco-benefits, as well as the personal moral benefits of staying in an environment-friendly green hotel. This could also include guests being provided with documentation and video links of non-green hotels and the negative effects these cause to the environment, focusing on pollution and resource wastage. Using these tactics may improve Generation Z tourists’ moral obligations and thus, enhance their attitude and intention to stay in green hotels.

Last, interest in green hotels has increased in recent years, but most green hotels in Taiwan are only involved in limited green hotel practices and do not provide enough information. As suggested by Verma et al. (2019), marketers should alter the way they promote their goods to raise tourist responsibility. For instance, changing the phrase from “*choose green hotels*” to “*choose a green hotel today; the planet will be saved tomorrow* “will enhance interest in green hotels. It might be a way to encourage visitors to feel strongly responsible. These actions can assist in increasing consumer environmental awareness and fostering a favorable attitude regarding green hotels in the future direction.

## Conclusion

Endeavors in this research contributed to a better understanding of the reasons and psychological mechanisms where the Z-generation tourists visit green hotel intention. This research result recommends and illustrates that TPB as well as PMN and EC, as an advanced integrative approach, can predict Z-generation tourists’ green hotel visit intention in Taiwan. Empirically findings confirm that AT, SN and PBC influence tourists’ green hotel visit intention. In addition, the findings supported earlier literature evidence of the impact of SN, PMN, and EC on the attitude toward green hotels. Surprisingly, however, the study also found an insignificant relationship between PMN, EC, and tourists’ intention toward green hotels. Furthermore, our research emphasized the mediator (as attitude) for understanding Z-generation tourists’ green hotel visit intentions. Notably, our findings reveal that AT play a crucial role in establishing a relationship between SN, PMN, and EC and their green hotel visit intention.

This research extends the current knowledge and provides an empirical model for tourism industry. Considering the major role of the tourism industry, our model is an important result of guiding the important role of visiting intention in hotel operations. This model can help hotel managers better understand the relationship between PMN, EC, TPB domain (AT, SN, PBC), and green hotels visit intention, which is a very important management tool.

The results should be interpreted while considering the limitations of this study. First, despite the substantial amount of research that utilizes self-reported behavior to measure Generation Z tourists’ green hotel visit intention, some scholars argue that consumers exaggerate their intentions to engage in pro-environmental behavior (Mahardika et al., 2020), for example, their green hotel visit intention. In the future, researchers may consider sampling actual behavior instead of using self-reported behavior intention.

Another limitation in this study is methodological. The suggested model was only tested with a limited sample. In future studies, it would be beneficial to test samples from different nations to validate the generalizability of the research model.

It is also recommended that future research distinguishes green hotel categories, such as luxury hotels, leisure hotels, budget hotels, business hotels, and Airbnb, as consumer expectations may differ depending on the type of green hotel. Finally, future research could consider other situational factors that could affect Generation Z tourists’ intention to visit green hotels, for example, the purpose of their trip, budget constraints, and/or the number of traveling companions.

## Data availability statement

The original contributions presented in the study are included in the article/Supplementary material, further inquiries can be directed to the corresponding author.

## Ethics statement

Ethical review and approval was not required for the study on human participants in accordance with the local legislation and institutional requirements. Written informed consent to participate in this study was provided by the participants’ legal guardian/next of kin.

## Author contributions

JP, Y-MT, and K-SW: conceptualization. T-CW: data curation. K-SW and T-CW: formal analysis, software, and writing—original draft. Y-MT: funding acquisition. JP and K-SW: methodology. JP and T-CW: project administration. JP, Y-MT, K-SW, and T-CW: writing—review and editing. All authors contributed to the article and approved the submitted version.

## Funding

This study is supported by “The 2021 I Offer Good Strategies for the Construction of New Fujian (United Front Special Project)”-JAT21111 Building a Green Accommodation System Based on Fujian’s New Ecological Province Strategy.

## Conflict of interest

The authors declare that the research was conducted in the absence of any commercial or financial relationships that could be construed as a potential conflict of interest.

## Publisher’s note

All claims expressed in this article are solely those of the authors and do not necessarily represent those of their affiliated organizations, or those of the publisher, the editors and the reviewers. Any product that may be evaluated in this article, or claim that may be made by its manufacturer, is not guaranteed or endorsed by the publisher.
