# Supplementary material for: Anticipating Z-generation tourists’ green hotel visit intention utilizing an extended theory of planned behavior
Source: Front Psychol. 2022 Dec 5;13:1008705. doi: 10.3389/fpsyg.2022.1008705 (PMC9764080; doi:10.3389/fpsyg.2022.1008705)
Supplement: Supplementary file 1 [file Table_1.docx]

Appendix 1. Survey items related to behavioral intention toward the green hotel.

| Variable | Item | References |
| --- | --- | --- |
| Personal moral norms (PMN) | | |
| PMN1 | Staying at a green hotel and using environment friendly products/services would make me a better person | Choi et al. (2015) |
| PMN2 | Instead of a conventional hotel, staying at green hotel will make me feel like a morally obliged person |  |
| PMN3 | Saving the environment should be the first priority for a person like me |  |
| PMN4 | Regardless of what other people do, I feel staying at green hotels is a moral obligation |  |
| PMN5 | Saving energy as much as possible is my personal obligation |  |
| Environmental concerns (EC) | | |
| EC1 | The balance of nature is very gentle and can be easily upset | Abdul-Muhmin (2007); Cordano et al. (2011) |
| EC2 | Human are severely abusing the environment |  |
| EC3 | Humans must maintain the balance with nature to survive |  |
| EC4 | Human interferences with nature often produce disastrous consequences |  |
| Attitude (AT) | | |
| AT1 | For me, staying at a green hotel when traveling is good | Kim and Han (2010); Teng et al. (2015); Verma & Chandra (2018) |
| AT2 | For me, staying at a green hotel when traveling is desirable |  |
| AT3 | For me, staying at a green hotel when traveling is pleasant |  |
| AT4 | For me, staying at a green hotel when traveling is favorable |  |
| AT5 | For me, staying at a green hotel when traveling is enjoyable |  |
| Subjective norms (SN) | | |
| SN1 | Most people who are important to me think I should stay at a green hotel when traveling | Chen & Tung (2014); Han et al. (2010); Kim and Han (2010) |
| SN2 | Most people who are important to me would want me to stay at a green hotel when traveling |  |
| SN3 | People whose opinions I value would prefer that I stay at a green hotel when traveling |  |
| Perceived behavioral control (PBC) | | |
| PBC1 | I am confident that if I want, I can stay at a green hotel when traveling | Chen & Tung (2014); Han et al. (2010); Kim and Han (2010) |
| PBC2 | Whether or not I stay at a green hotel when traveling is completely up to me |  |
| PBC3 | I have resources, time, and opportunities to stay at a green hotel when traveling |  |
| Intention (INT) | | |
| INT1 | I am willing to stay at a green hotel when traveling | Chen & Tung (2014); Han et al. (2010); Kim and Han (2010) |
| INT2 | I plan to stay at a green hotel when traveling |  |
| INT3 | I will make an effort to stay at a green hotel when traveling |  |
